# Wearable Devices for Assessing Function in Alzheimer's Disease: A European Public Involvement Activity About the Features and Preferences of Patients and Caregivers

**DOI:** 10.3389/fnagi.2021.643135

**Published:** 2021-04-12

**Authors:** Thanos G. Stavropoulos, Ioulietta Lazarou, Ana Diaz, Dianne Gove, Jean Georges, Nikolay V. Manyakov, Emilio Merlo Pich, Chris Hinds, Magda Tsolaki, Spiros Nikolopoulos, Ioannis Kompatsiaris, The RADAR-AD Consortium

**Affiliations:** ^1^Center for Research and Technology Hellas (CERTH-ITI), Information Technologies Institute, Thessaloniki, Greece; ^2^Medical School, Aristotle University of Thessaloniki (AUTH), Thessaloniki, Greece; ^3^Alzheimer Europe (AE), Luxembourg City, Luxembourg; ^4^Clinical Insights and Experience, Janssen Research and Development, Beerse, Belgium; ^5^Alfasigma Schweiz AG, Zofingen, Switzerland; ^6^Big Data Institute, University of Oxford, Oxford, United Kingdom; ^7^Greek Association of Alzheimer's Disease and Related Disorders (GAADRD—Alzheimer Hellas), Thessaloniki, Greece

**Keywords:** Alzheimer's disease, dementia—Alzheimer disease, wearable sensors devices, public involvement, caregivers, technology acceptance and perception, technology acceptance and adoption, internet of the things

## Abstract

**Background:** Alzheimer's Disease (AD) impairs the ability to carry out daily activities, reduces independence and quality of life and increases caregiver burden. Our understanding of functional decline has traditionally relied on reports by family and caregivers, which are subjective and vulnerable to recall bias. The Internet of Things (IoT) and wearable sensor technologies promise to provide objective, affordable, and reliable means for monitoring and understanding function. However, human factors for its acceptance are relatively unexplored.

**Objective:** The Public Involvement (PI) activity presented in this paper aims to capture the preferences, priorities and concerns of people with AD and their caregivers for using monitoring wearables. Their feedback will drive device selection for clinical research, starting with the study of the RADAR-AD project.

**Method:** The PI activity involved the Patient Advisory Board (PAB) of the RADAR-AD project, comprised of people with dementia across Europe and their caregivers (11 and 10, respectively). A set of four devices that optimally represent various combinations of aspects and features from the variety of currently available wearables (e.g., weight, size, comfort, battery life, screen types, water-resistance, and metrics) was presented and experienced hands-on. Afterwards, sets of cards were used to rate and rank devices and features and freely discuss preferences.

**Results:** Overall, the PAB was willing to accept and incorporate devices into their daily lives. For the presented devices, the aspects most important to them included comfort, convenience and affordability. For devices in general, the features they prioritized were appearance/style, battery life and water resistance, followed by price, having an emergency button and a screen with metrics. The metrics valuable to them included activity levels and heart rate, followed by respiration rate, sleep quality and distance. Some concerns were the potential complexity, forgetting to charge the device, the potential stigma and data privacy.

**Conclusions:** The PI activity explored the preferences, priorities and concerns of the PAB, a group of people with dementia and caregivers across Europe, regarding devices for monitoring function and decline, after a hands-on experience and explanation. They highlighted some expected aspects, metrics and features (e.g., comfort and convenience), but also some less expected (e.g., screen with metrics).

## Highlights

**What was already known about this topic**

- Remote monitoring technologies are promising to improve the current care of people with dementia in several aspects, such as timely assessment and intervention.
- The adoption and acceptance of technology by people with dementia is challenging.
- Several studies have investigated potential barriers to the adoption of the proposed health remote technologies through phone interviews and online questionnaires.

**What this study added**

- The Public Involvement (PI) activity included focused discussion, hands-on experimentation and detailed presentation of several candidate devices for people with dementia and their caregivers.
- Members of the RADAR-AD Patient Advisory Board (PAB) were given tools specifically designed to rate and rank the various features of the devices presented to them, by order of preference, as well as metrics and aspects of devices in general.
- Each device comes with its own peculiarities and combination of features, so people with dementia and their caregivers need to drive the selection process of the devices to be used in clinical research and future trials, including the RADAR-AD project study.

## Introduction

Current estimates suggest that there are around 9 million of people living with dementia across Europe (Alzheimer Europe, 2020) of which the most prevalent one is Alzheimer's Disease (AD) dementia. In addition, the current conceptualization of AD has been extended to encompass the full spectrum of the disease, including both pre-dementia, i.e., preclinical and prodromal AD or Mild Cognitive Impairment (MCI) due to AD, and dementia phases (Alzheimer's dementia) (Alzheimer Europe, 2020). An important aspect of the diagnosis, in AD and other dementias, is functioning, where current assessment methods rely mostly on self-report and observation by the caregivers. While this information is important, it requires considerable effort and time and still may be inaccurate. Therefore, existing traditional monitoring methods could be complemented by remote, objective, non-intrusive and relatively effortless monitoring, using technology.

### Technology for Older Community-Dwelling People With Dementia

The absence of objective data to assess the daily function of people with dementia could be addressed by several advances in digital technology for monitoring and analysis. Objective remote monitoring using digital technology could complement existing methods and lead to more accurate and timely assessment as well as more efficient clinical trials, proposing more effective interventions and ultimately improving both short and long- term care. Developing remote monitoring solutions and adapting them to the needs of people with dementia and their caregivers could, therefore, allow them to live independently at home for longer, support their caregivers and support decisions of healthcare professionals easily and timely, while promoting “aging in place” (American Planning Association and the National Association of County and City Health Officials, 2009).

Remote monitoring technologies (RMTs) can include one or many of the following components: smartphones, Apps, Internet of Things (IoT) sensors, both wearable and ambient smart home solutions, biomedical devices coupled with analytics. Smartphones, can help assess social behavior, via monitoring calls, text messages, or internet browsing, since mobile phone usage by elders is increasing (Anderson and Perrin, 2017), as do the applications of smartphones for health (Joe and Demiris, 2013). Meanwhile, wearable smart devices and remote health monitoring solutions have also been increasing in popularity over the last decade, especially for elders in general and people with dementia in particular (Lazarou et al., 2016, 2019; Megges et al., 2018). More specifically, smart home healthcare solutions can significantly delay nursing home admission (Kim et al., 2017) and promote safety monitoring and care of older adults through mobile devices (Albert et al., 2012; Lazarou et al., 2016, 2019), wearables (Carrino et al., 2012; Al-Shaqi et al., 2016) and other types of sensors (Mahoney et al., 2007; Mihailidis et al., 2008; Aloulou et al., 2013; Hawley-hague et al., 2014; Stucki et al., 2014; Piau et al., 2015; Lazarou et al., 2019). Smartwatches and wristbands are blooming in the electronics retail market. Their purpose primarily is to monitor daily activity and lifestyle including movement and sleep, in order to promote health and well-being. More research-oriented devices can measure activity levels, stress, heart rate, gait and other vital signs.

### User Acceptance of Technology

While technology seems to be a promising solution, its adoption is often challenging for end-users, especially older people and healthcare professionals. Technology experts tend to select devices based only on their desire to record the most appropriate signals with the highest granularity and precision. However, the same devices might be quite uncomfortable, heavy and too complicated for users, diminishing the outcome and the success of a study, which is particularly important in the case of people with dementia. Thus, the technology selection process should involve both people with dementia and their caregivers, who will daily operate such technologies. The number of different devices and possibilities increases every year, resulting in a variety of factors that might affect developers, such as data heterogeneity, manufacturer communication standards and programming interfaces, but also the end-users, such as shapes, materials, battery life, design, functionality, precision, and range (Boll et al., 2018). All these parameters should be considered, when selecting proper devices for monitoring users or for examining the potential adoption from them, especially in the case of treating people with AD. Thus, the feedback from the people with dementia as well as their caregivers introduces a valued end-user perspective in the selection process, with parameters that might otherwise be overlooked.

Older people, their family and caregivers can face considerable stress with the newly introduced technological components (Laguna and Babcock, 1997; Dyck et al., 1998; Tung and Chang, 2007). Most of the time, older adults express technology-related concerns, while the perceived benefits of technology might be more abstract to them. The most common barriers in the adoption of technology by older people are: familiarity and access, need for assistance, trust, privacy implications, design, reduced dexterity, precision, and physical issues (Fischer et al., 2014; Peek et al., 2014; Khosravi and Ghapanchi, 2015; Liu et al., 2016; Yusif et al., 2016; Zhao et al., 2018; Alshahrani et al., 2019). On the other hand, the most highlighted benefits of technology used by older people are: safety, perceived usefulness, independence, and reduced “burden” on family and caregivers (Peek et al., 2014). A crucial component to integrate and accept technology in real-life situations (e.g., at home) is to design and develop user-friendly user interfaces (UIs), to facilitate user interactions with the system (Liu and Yang, 2014). In this direction, the interaction of users with technology that takes into account their concerns and preferences has been shown to empower and engage them more in their care (Villalba-Mora et al., 2015).

### Existing Explorative Studies for Technology Adoption

Several exploratory studies have investigated potential barriers in the adoption of RMTs through constructive questionnaires. A recent study, using Theory of Planned Behavior (TPB), explored the potential intention of adult children to use online health information for their aging parents (Bao et al., 2017), and its findings showed that they are willing to use such technological solutions. Another study, using TPB, investigated the potential adoption of mobile health services (Deng et al., 2014) and found that perceived value and behavior control, resistance to change and attitude can be precursors for using mobile health services for the middle-aged group, while additional traits such as self-actualization need and technology anxiety found to affect the behavior intention of older participants. An exploratory study examined the attitude and acceptance of women in Singapore, above 50 years of age, toward a mobile phone-based intervention through a phone survey (Xue et al., 2012). They found that the women were likely to adopt the proposed solution if it was considered as useful and easy-to-use. A questionnaire survey identified that the main factors that affect the acceptance of health technology by people with chronic conditions were: attitude toward technology, perceived usefulness, ease of learning and availability, social support, and perceived pressure (Sun and Rau, 2015). Another paper-based questionnaire survey found that the most important factors underlying the acceptance of technology by older adults were: satisfaction, perceived usability, support availability, and public acceptance (Wang et al., 2011). Furthermore, in (Wong et al., 2012), the authors evaluated the user's intention to use different systems for elders (i.e., the Medication Reminder, Dr. Ubiquitous, Sharetouch, and Intelligent Watch), by administering a modified technological acceptance model (TAM) questionnaire. The participants who used the Intelligent Watch showed the greatest willingness and satisfaction, while the ones who used Dr. Ubiquitous revealed little eagerness regarding the perceived ease of use. More long-term studies investigated commercially available RMTs (Giger et al., 2015), showing that the developed TAM questionnaire revealed that the elders as well as their caregivers and their friends responded positively regarding acceptance. Finally, another study explored attitudes and perceptions of contactless ADL monitoring across 15 older people, and found that they would easily integrate the suggested technology into their daily life (Claes et al., 2015). However, various concerns were outlined related to the operation and the pricing of the proposed contactless monitoring technology.

### Commonalities and Differences

In general, exploratory studies so far have focused on the adoption of the suggested technological solutions by older people and their caregivers without presenting them the device characteristics and particular features in detail. Yet, an earlier review study encouraged professionals and caregivers to describe concrete benefits and technological advances to the older people in order to minimize technology-related concerns, while also giving them the opportunity to try out the technology in a risk-free environment (Peek et al., 2014). Another study clearly states that ease of use cannot really be self-reported and actual use is hard to be determined, since the participants cannot conceptualize and visualize themselves using the technology unless they have used it before (Xue et al., 2012). The study concludes that “it may be more enlightening to observe users through focus groups, by trying out a prototype interface.”

Based on that, the present Public Involvement (PI) activity involves people with dementia and their caregivers who are members of a Patient Advisory Board (PAB). It also employs a hands-on approach where different solutions are demonstrated, presented in detail with respect to features and offered to participants to test them before collecting feedback. Furthermore, most studies explore either a single demonstrated technology or general features and preferences, whereas this PI activity is part of a device selection process, where several candidate devices were presented. Moreover, in contrast to most studies, the group included participants from several countries from Europe who are members of a PAB of a large European IMI-funded project. The PAB members are men and women with different types of dementia, different stages and experiences, and their caregivers. The composition of the PAB can be found online^1^.

Furthermore, in comparison with the exploratory studies so far, most of which describe technology to participants via brochures, online questionnaires or verbally, the present study gives the opportunity to participants to experience and feel the devices hands-on in order to better understand and prioritize particular features based on their preferences. Additionally, the majority of exploratory studies have been focused on the older age dwelling population in general and not particularly on people with dementia. Also, in most studies participants completed the questionnaires themselves (e.g., via telephone or given a paper-pencil questionnaire) without someone being present and explaining possible questions. Another drawback of existing surveys is that they mainly provide a general description of several multi-purpose technological devices (e.g., PC, digital camera, video recorder, and mobile phone) without including tailored questions for a particular type of a device, applications and features of it, relying the answers solely on the appearance and the practical use of them.

### Aim of the PI Activity

The aim of this PI activity is to guide the decision about device selection for the wearables to be used in the clinical trials of the RADAR-AD project^2^. The RADAR-AD project aims to improve the assessment of AD through digital biomarkers extracted from the use of smartphones, wearables, and smart home sensors with respective apps and analytics tools. The RADAR-AD trials focus on remote assessment of people with dementia while at the same time offering support to their informal caregivers. Tier 1 of the study focuses on using wearable devices continuously throughout the day and several apps during the observational period and at baseline and last visit to clinic. More specifically, our study examined several diverse wearables and considered also the acceptance or not of particular devices through an open session involving both people with dementia and caregivers.

This paper focuses on understanding the unique preferences, needs and concerns of people with dementia and caregivers at the core of a device selection process for a wearable in the framework of the RADAR-AD trials. The selection of wearables presented to the users is not limited only to specific devices available in the market nor are the features and preferences extracted from them. As such, it aims to serve as a guide for any future trial involving people with dementia and caregivers.

**Figure 1:**
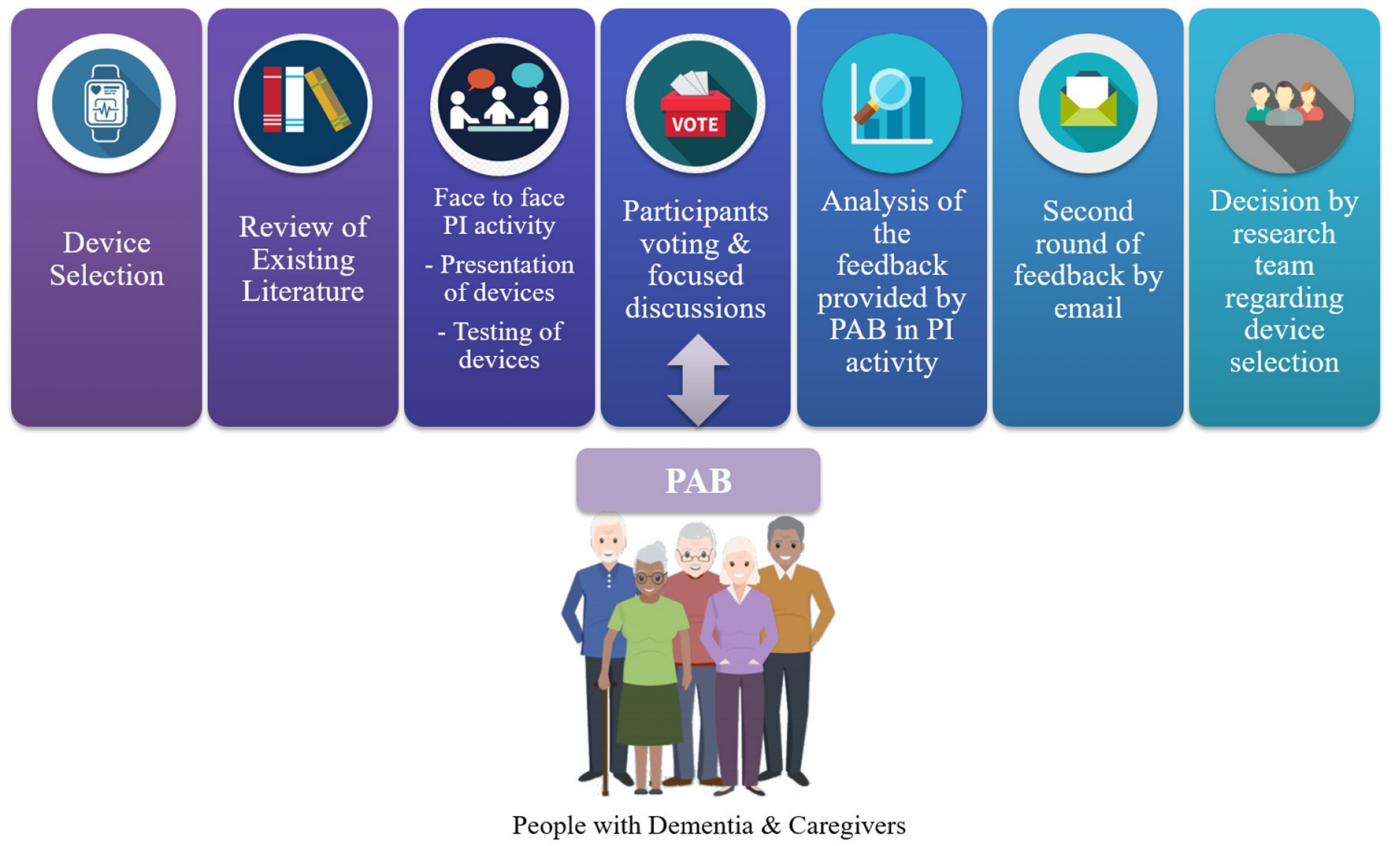
The framework of the study centered around people with dementia and their caregivers.

The process for the PI activity and its steps are illustrated in Figure 1. People with dementia and caregivers first highlighted particular features tailored to the disease, memory, and other functional impairments, as, for example, the necessity for a waterproof device or a sound or blinking light notification to remind users to charge the device. Then, an optimization process identified the best compromise between the most technologically advanced and user-accepted devices to satisfy both parties. Thus, the present PI activity described in this paper explored some of the potential concerns and requirements relevant to both people with dementia and their caregivers through a user-centered approach. Additionally, we examined four specific brands/models of wearables and deducted results about participants' preferences.

This paper is structured as follows. In section Introduction, we present a general background about the concept of health-related technologies and other studies in the field of technology adoption from older adults. Section Materials and Methods provides details on the PAB PI activity and the presentation and feedback collection process designed for this work. Section Results presents the results and descriptive statistics, while section Discussion considers discussion, limitations, comparisons of results with similar approaches, and suggestions for future research. Finally, section Conclusion presents conclusions drawn.

## Materials and Methods

### Participants and Setting

All members of the PAB were members of the European Working Group of People with Dementia (EWGPWD) European Working Group of People with Dementia - Alzheimer Europe, which comprises of people from different countries across Europe. Its members have been diagnosed and informed of their diagnosis^3^ and play an active role in collaborative research projects, such as in RADAR-AD. The members of the EWGPWD had all agreed to be members of the RADAR-AD PAB and will be referred to hereafter as PAB members. The PAB was a diverse group composed of 11 people with different kinds of mild to moderate dementia (mainly Alzheimer's dementia, one person with frontotemporal and one person with vascular dementia) and 10 carers from 11 different countries, namely, from the Czech Republic, Bosnia and Herzegovina, the Republic of Ireland, England/Wales, Scotland, Portugal, Belgium, Sweden, Finland, Austria, and Germany.

Given that the PAB involves members of the public in the design and development of research to act as advisers, providing valuable knowledge and expertise based on their experience of a health condition or as a carer/caregiver, the PI activity does not raise ethical concerns and, thus, does not require ethical approval, according to the National Health Services (NHS) Health Research Authority^4^. In detail, and with respect to those guidelines: (i) the PAB involves members of the public for assurances on aspects of the design of future research, making it more relevant to the people it is trying to help, helping to define what is acceptable to participants and improving their experience; (ii) the PI activity involves the PAB in the research process with or by the public and not to, about or for them; and (iii) the public is involved in identifying and prioritizing research topics, plays the role of a research advisory group, identifies outcome measures which are meaningful and relevant to patients and comments on the feasibility of the research design including the burden of participants and the levels of risk/distress they may be exposed to.

The PI activity took place during the first RADAR-AD PAB meeting in Luxemburg on 18 April 2019. In total, 21 (*N* = 21) PAB members took part. In order to present the devices, support, and collect feedback more efficiently and effectively, the PAB was divided into three groups—round tables of seven people and one facilitator—researcher. The three groups were at the same room and were asked the same questions/tasks. The three facilitators were researchers, familiar with the technology and applications in AD. Two members of Alzheimer Europe supported all three groups when needed. We asked all PAB members whether they wished to take part in the session and gave them verbal and written information about the activity prior to the meeting. All agreed to participate in this PI activity, with anonymity and confidentiality by all researchers. The input provided by the participants during the activity was anonymized and stored locally (offline).

The researchers explored a vast number of lifestyle wearables and sports wearables available in the market as well as some more research-oriented wearabels, paying particular attention to a range of characteristics such as their size and weight (e.g., from light and comfortable size to heavier), more feature-rich alternatives (e.g., several icons and choices), accuracy and battery life. For the purpose of the PI activity, four devices were selected which represented four different combinations of those parameters. To minimize possible biases participants did not know the marketed names of the four devices (they were refered to as Bracelet 1, 2, 3, and 4). The accuracy of the devices was known from previous experimental evaluation studies (Stavropoulos et al., 2020). Being representatives means that some devices can be replaced in the future with alternatives of similar properties and features. The selected devices were:

- **Bracelet 1—**measures steps, sleep and heart rate (HR), high accuracy, light, high comfort, 7-day battery life, monochrome touchscreen, and waterproof
- **Bracelet 2—**measures steps, sleep, HR and 3D movement (XYZ accelerometer), high accuracy, bulkier, soft, 2-day battery life, and color touchscreen
- **Bracelet 3—**measures steps and sleep, average accuracy, the lightest (is a wristband), 7-day battery life, and no screen
- **Bracelet 4—**measures steps, sleep, HR, heart rate variability and perspiration (Galvanic Skin Response—GSR), high accuracy, large and heavy, 1-day battery life, and no screen

### The PAB Session

**Figure 2:**
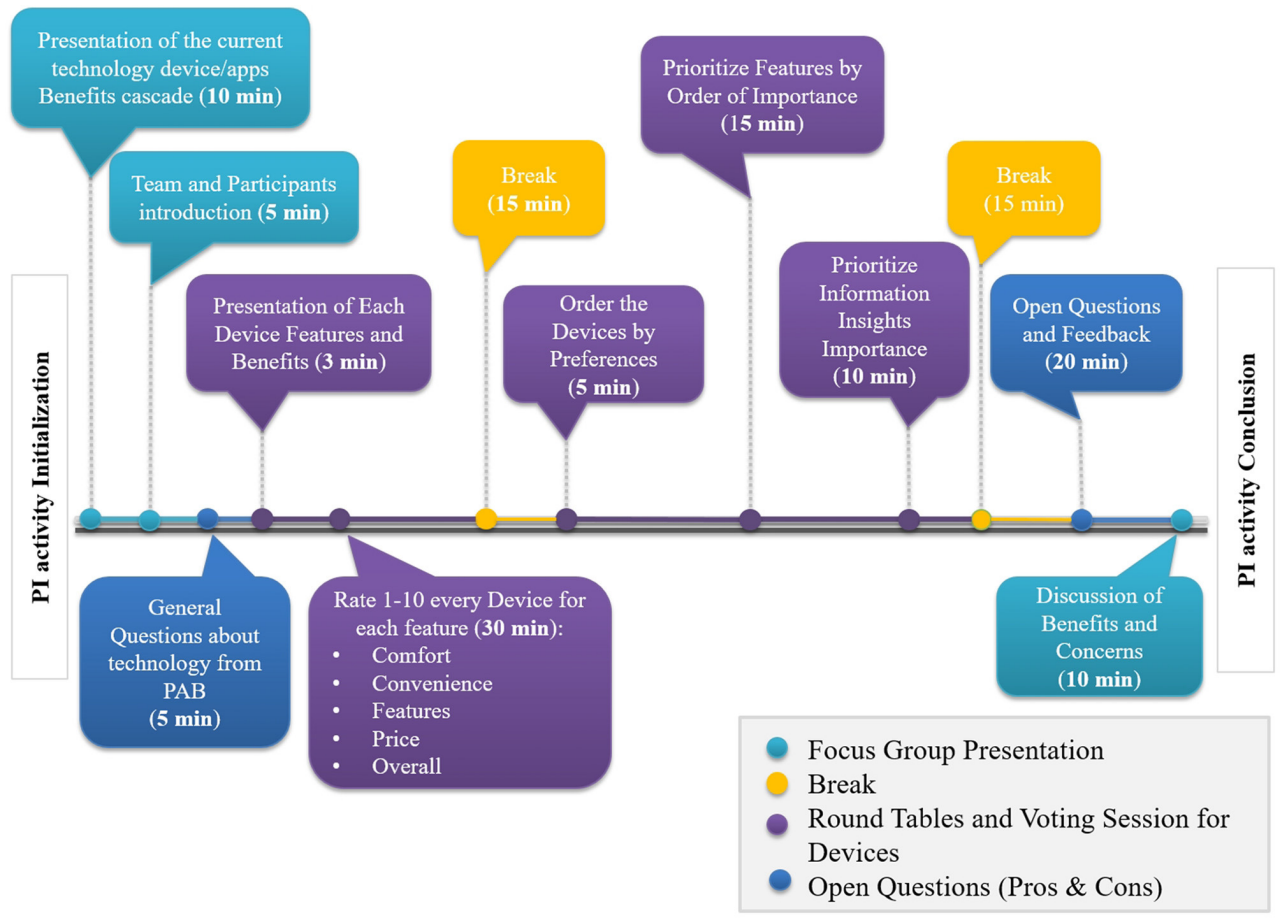
Focus Group process timeline.

**Figure 3:**
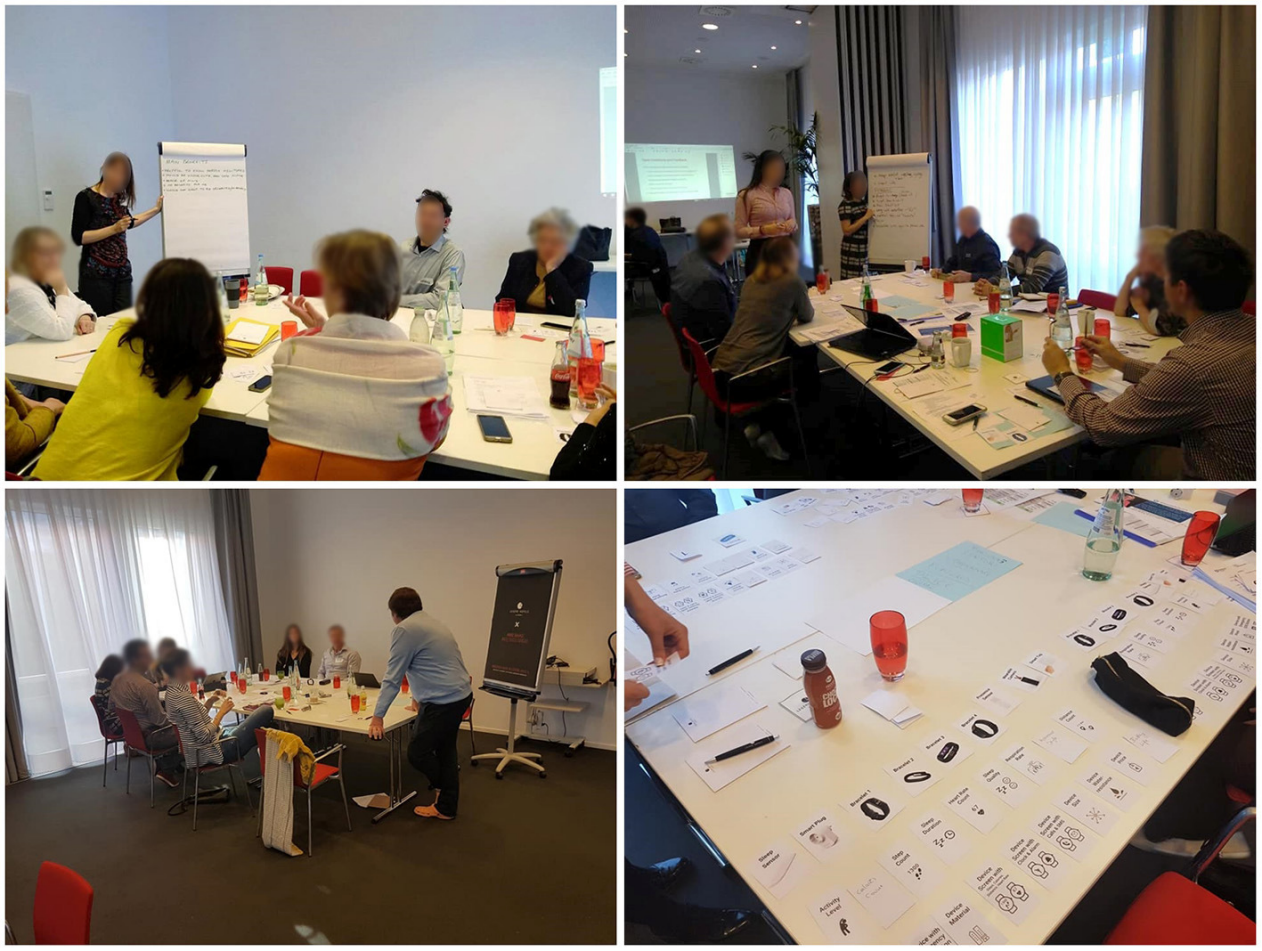
The setting, the three groups—round tables and materials used during the PAB Session.

The total duration of the PI activity was around two and a half hours, including a structured voting session and close questions which were quantitatively analyzed as well as general and open discussion. The structure and timeline of the session is illustrated in Figure 2. Each activity block is presented in the following subsections. Additionally, Figure 3 shows the setting, the groups and the materials used during the PAB session. The PAB session was organized as follows:

- Introduction to the RADAR-AD project, the PI aim, current technology status of devices/apps and their potential benefits for the researcher, the clinician and the user
- Presentation and rating of four specific wearable devices and their features
- Ranking devices in order of preference and prioritizing device-independent features and metrics
- Open discussion about the devices and their use

#### Presentation of Current Device Technology

Initially, the facilitator—researcher and members of Alzheimer Europe presented the general outline of the activity aims and the RADAR-AD study context. Then, the facilitators presented all current technology of the devices, while describing the potential benefits for the researcher, the healthcare professionals and the beneficiaries (people with dementia and caregivers) as described in the literature.

#### Examining and Evaluating Each Device Separately

**Figure 4:**
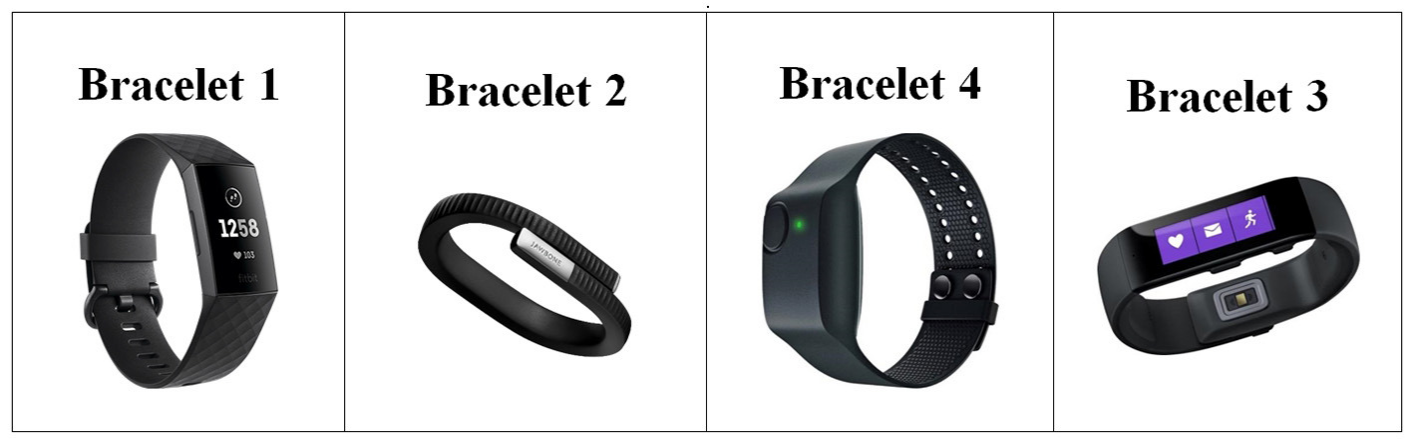
The four wearable devices provided to the participants, as they are depicted on the device card set.

Afterwards, a presentation on a projector was shown for each device, listing its main features for the user (battery life, screen, waterproof, etc.) and its value for researchers/clinicians/persons (metrics and accuracy). Then, 2–3 of each device were provided to the round tables for the PAB members to wear and test, e.g., by trying the touchscreen, checking the time and measurements of HR, steps etc. After the presentation of the devices, the facilitators answered questions raised by the PAB. Discussion followed for the pros and cons of the devices and more detailed feedback was given. For the voting, each participant was provided with a “device card set,” with device depictions printed in color, exactly as shown in Figure 4. This would help the participants remember the wearable devices when they are not physically present on their table.

Also, each person was given a “voting card set” with the numbers 1–10. In each voting round, votes were cast—card face-down on the table, counting 1–2–3 and then revealed simultaneously to avoid bias and contribute to fun. More specifically, the PAB was asked to rate every device for each feature from 1 to 10 with regards to (a) *Comfort* while wearing or living with the device at home (Feature: Size, Weight, and Material), (b) *Convenience* while charging it, taking it on and off etc. (Feature: Battery Life, Water-resistance, etc.), (c) *Features* for the user (Feature: Screen with Clock/Alarm, Calls, Steps/Sleep/Calories/HR), (d) *Price* if you were to buy it yourself and value-for-money, and (e) *Overall* device rating. The abovementioned rating was repeated for each of the four bracelets, yielding 20 rates per PAB member. The PAB was then asked why they voted as they did and to expand on the issues further. The total duration of this segment was around 50 min, followed by a 15 min break.

#### Cross-Device, Feature, and Qualities Ranking

**Figure 5:**
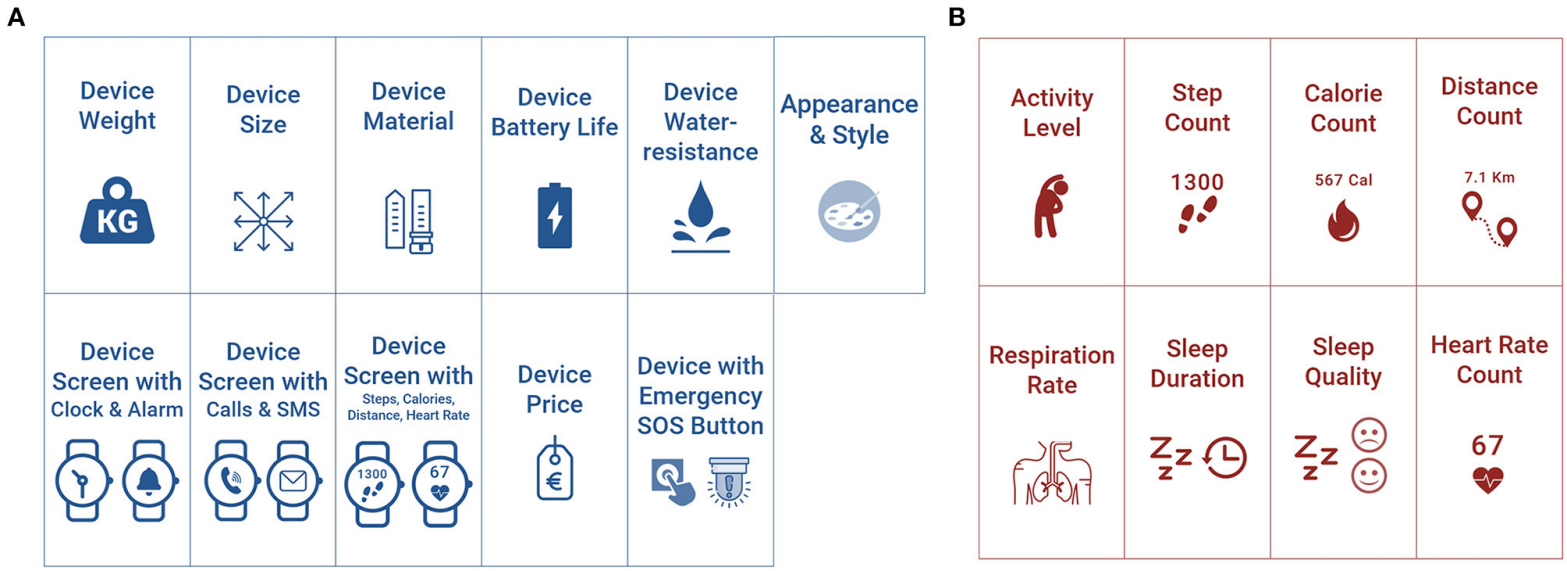
The feature **(A)** and the metrics **(B)** card sets.

The PAB was asked to rank devices, features, and qualities by arranging the respective card sets from left to right accordingly (left: most preferable, right: least preferable). Firstly, they were asked to order the four wearable devices from the previous segment, by overall preference. Then, a new card set was introduced to examine device features in general, independent of specific device implementations. They were then asked to prioritize the device “feature card set” (Figure 5A) from those most to those least important to them: Weight, Size, Material, Battery Life, Water-resistance, Screen with Clock/Alarm, Screen with Calls/SMS, Screen with Steps/Calories/Distance/Sleep/HR, Appearance matching your taste/style, Emergency/Panic Button to call help, and Price. Finally, the “metrics card set” (Figure 5B) was introduced so that they could prioritize the various types of measurements that can potentially be provided by devices from most to least important to them (Physical Activity Level, Steps, Calories, Distance, Heart Rate, Respiration Rate, Sleep Duration, Sleep Quality—Light, Deep). The total duration of this segment was around 30 min, followed by a 15 min break.

#### Open Questions and Feedback

Finally, all PAB members in their round tables had the opportunity to discuss and elaborate about the potential benefits and concerns of using these devices, according to their personal belief. Then the facilitators communicated the results of all the rankings to the PAB members.

## Results

### Device Selection

**Figure 6:**
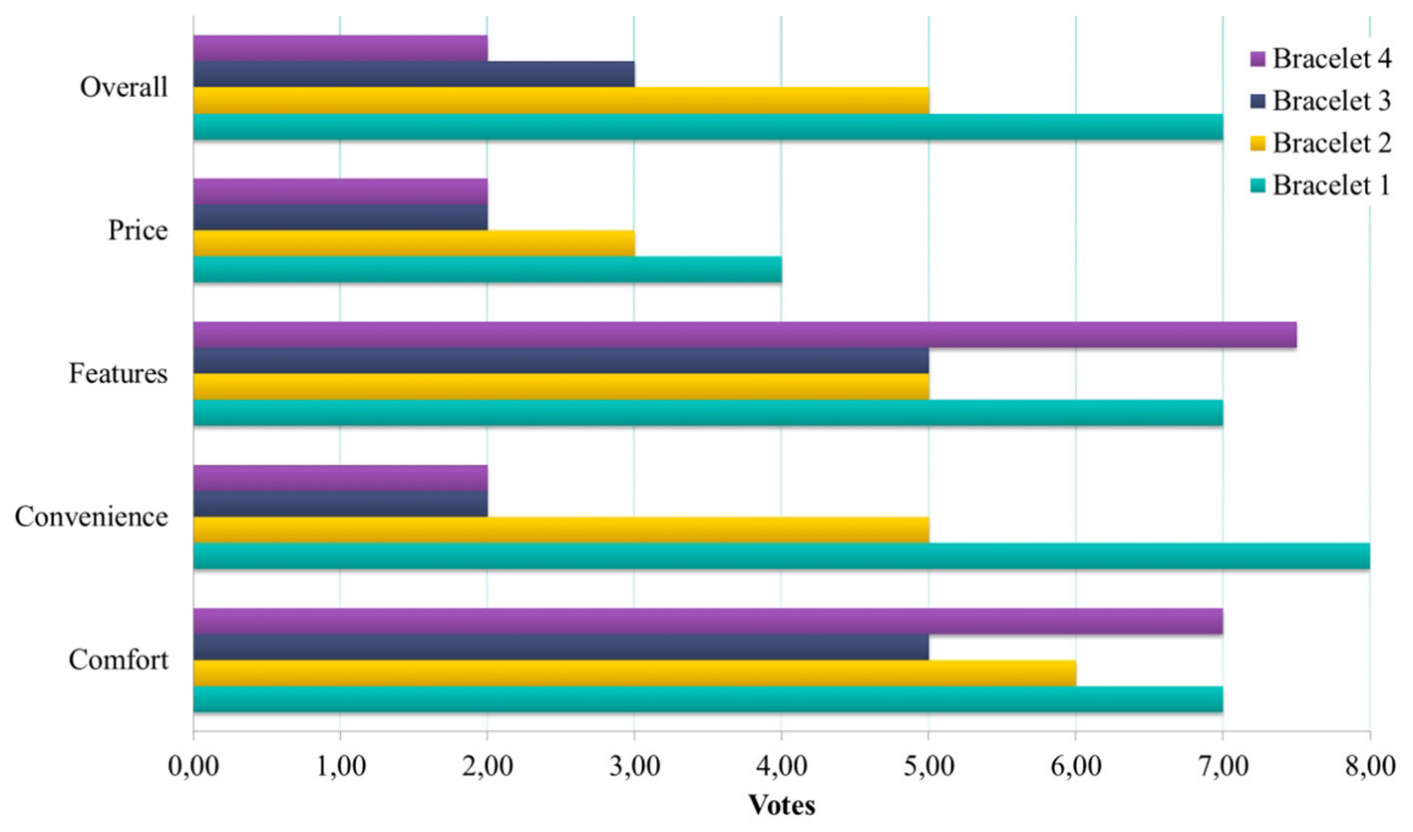
Average rating per device aspect for each of the four bracelets.

**Figure 7:**
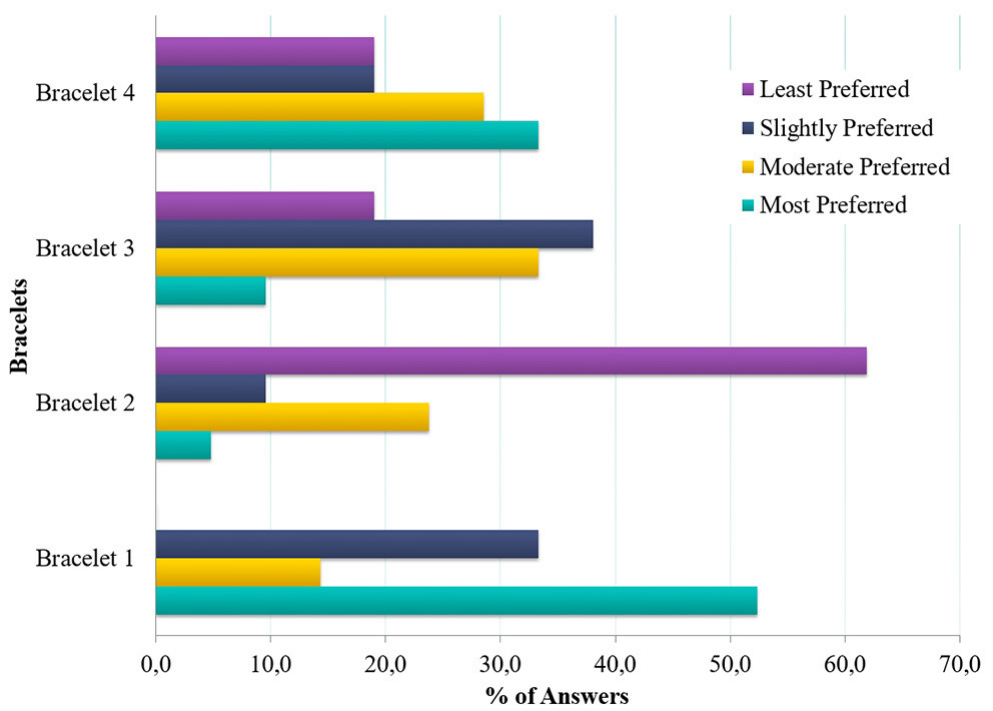
Preferred order of Bracelets: percentage of PAB members who placed each bracelet in each place in an order of preference from 1 (most preferred) to 4 (least preferred).

Regarding the particular device selection, the participants gave ratings (from 1 to 10) for each aspect and an overall rating for each device. Then they ordered the devices by preference. The results from the voting session are illustrated in Figure 6 (per Device ratings) and Figure 7, while mean values and standard deviation are presented in Table 1.

**Table 1:** Order of the devices based on preference (% of Answers) and rating per device (Mean and Standard Deviation).

	**Bracelet 1**	**Bracelet 2**	**Bracelet 3**	**Bracelet 4**
**Rating per Device—*****M*****(*****SD*****)**				
Comfort	7.00 (2.16)	6.00 (2.62)	5.00 (1.71)	7.00 (2.33)
Convenience	8.00 (2.31)	5.00 (2.04)	2.00 (1.20)	2.00 (1.57)
Features	7.00 (2.35)	5.00 (1.74)	5.00 (1.71)	7.50 (2.89)
Price	4.00 (3.10)	3.00 (2.33)	2.00 (1.93)	2.00 (2.56)
Overall	7.00 (1.96)	5.00 (2.10)	3.00 (1.82)	2.00 (2.33)
**Order based on Preference—%**				
Most Preferred	52.4%	4.8%	9.5%	33.3%
Moderate Preferred	14.3%	23.8%	33.3%	28.6%
Slightly Preferred	33.3%	9.5%	38.1%	19.0%
Least Preferred	0.0%	61.9%	19.0%	19.0%

The PAB favored bracelets that they perceived as convenient, comfortable, and affordable and feature rich. The most high-rated bracelet (Bracelet 1) was also perceived as the most comfortable to wear, convenient (less charging) and affordable. A bracelet that is light to wear and convenient, but does not have a screen feature was less prefered (Bracelet 2). On the contrary the most feature-rich bracelet was the least prefered due to incovenience (frequent charging) and despite being comfortable (Bracelet 4).

Along those lines, most PAB members again selected the bracelet they perceived as the most comfortable, convenient and affordable with enough features (Bracelet 1). A bracelet that is light and comfortable but without a screen was the least preferred here, highlighting the importance for some indication features (Bracelet 2), explored in the next segment.

### Metrics and Features

**Figure 8:**
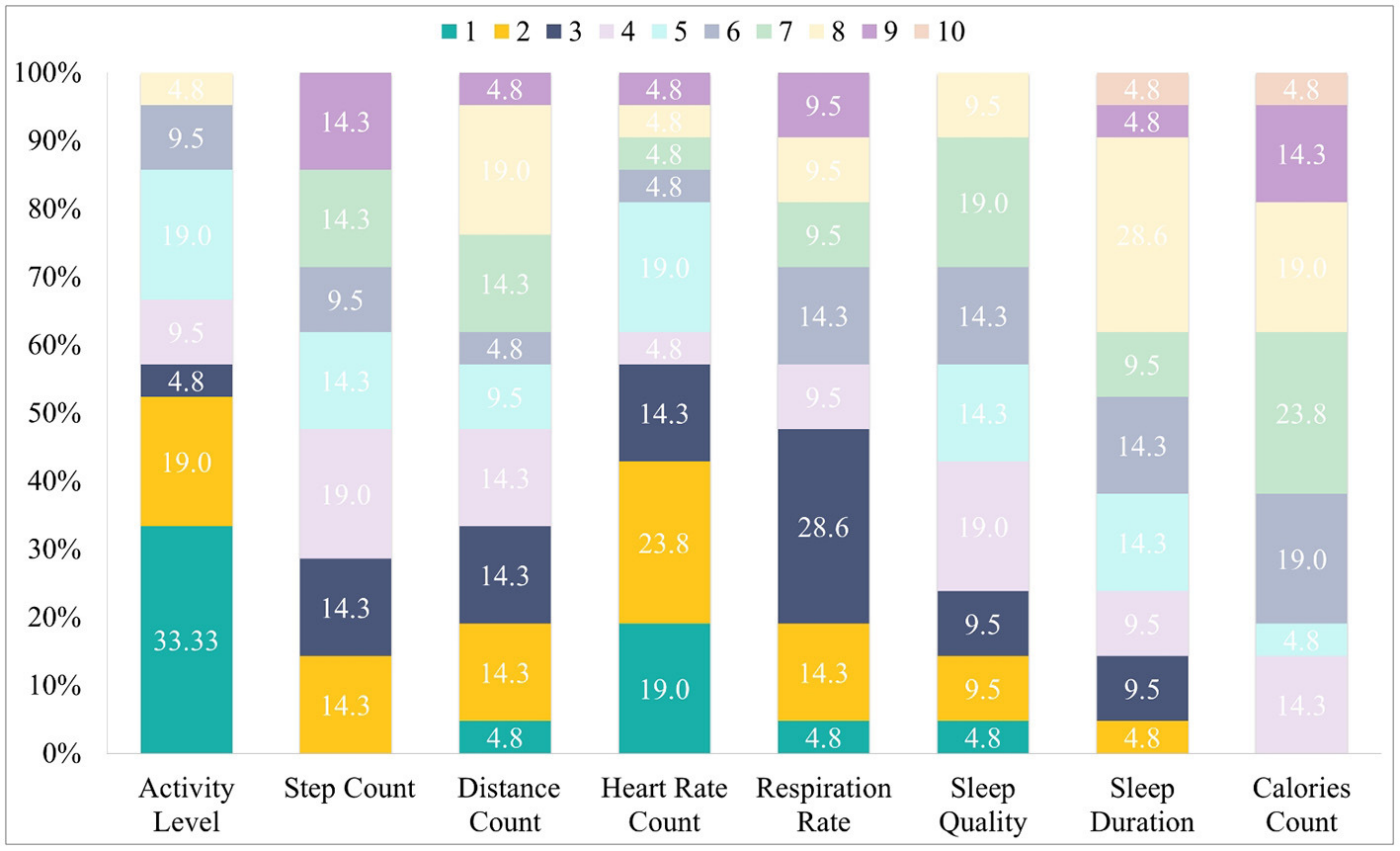
Preferred order of Metrics: percentage of members of the PAB that placed the metric in each place from 1 to 10 (most to least preferred).

Regardless of device, PAB members ranked “Activity Level” and “Heart Rate” as the most interesting and useful metrics a device could measure. These metrics were ranked in the first place by 33.3 and 19%, respectively. Also, these two metrics were ranked in the first, second, or third place by more than half of the PAB members. Other metrics which also received votes for first place but by fewer people included Sleep Quality, Distance Count and Respiratory Rate (ranked in the first place by 4.8% each). The full order of metrics is shown in Figure 8.

The most important feature of a candidate device was: “Appearance and Style,” which was ranked in first place by many PAB members (53.8%). This was followed by “Water-Resistance,” “Price,” and an “Emergency Button” (19% each), “Screen with Steps and Heart Rate metrics” (14.3%) and “Weight,” “Material,” and a “Screen with Calls and SMS” (9.5 %). Notably, “Battery Life” was ranked second by several PAB members (42.9%), followed by a “Screen with Clock and Alarm” and “Size” (14.3% each).

**Figure 9:**
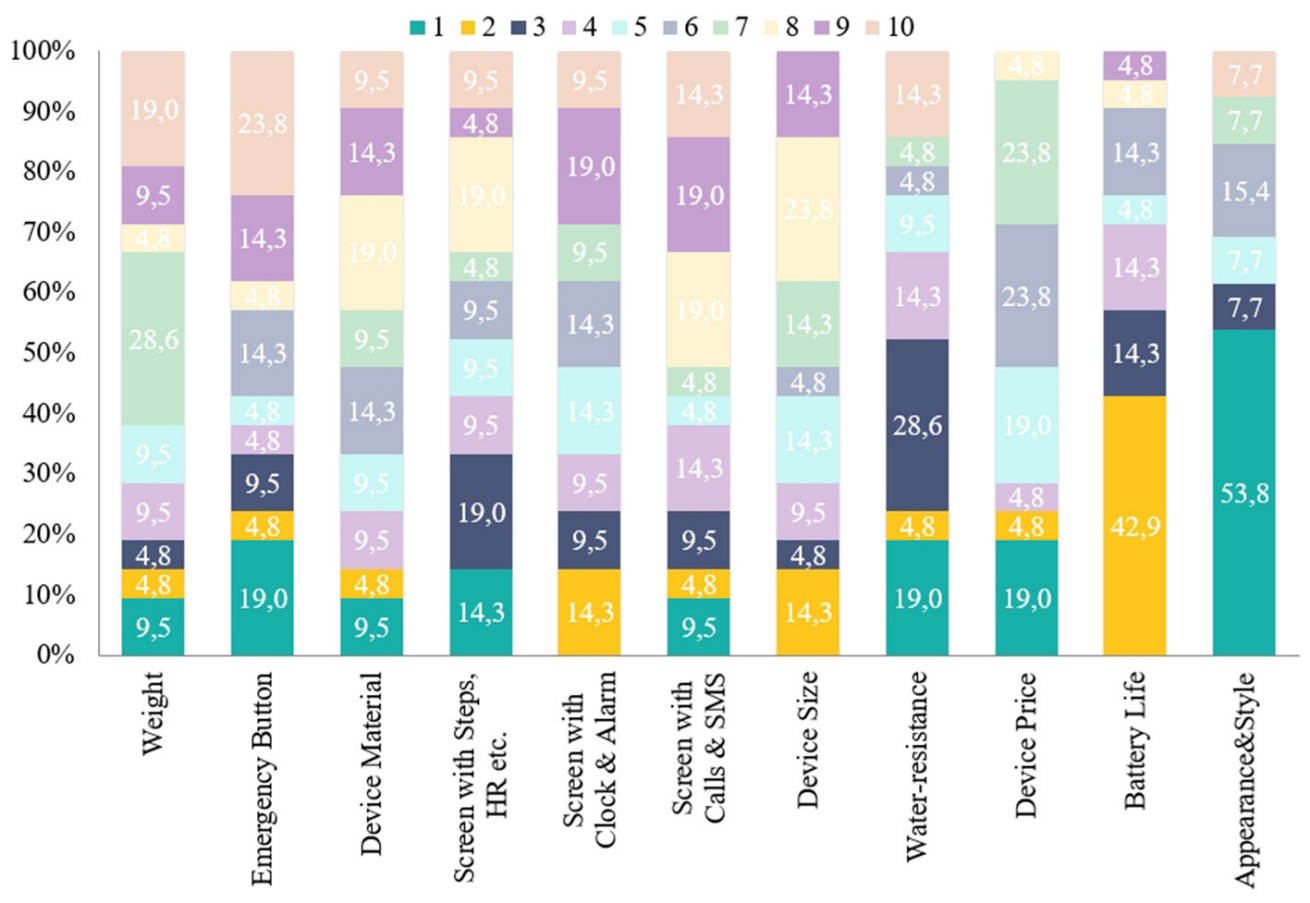
Preferred order of features: percentage of PAB members who placed each feature in each place from 1 to 10 (most to least preferred).

“Appearance and Style,” “Battery life,” and “Water resistance” were ranked in first, second, or third place by more than half of the PAB members. The full order of features is shown on Figure 9.

### Benefits and Concerns—Open Discussion

After the voting and ordering session, PAB members were asked about different existing devices and in particular about the most important aspects to consider when selecting a particular device. Relevant issues were linked to the devices being waterproof, easy to use, comfortable to wear and nice. Many felt the type of information or support the person could gain or have whilst using it was very important (e.g., information about their health such as sleep, heart rate, etc., information about where the person is such as tracking/GPS system, time, and date etc.). Concerns about the devices included that the person could forget to charge the devices, misplace it or lose it and the potential anxiety or distress if the person forgot how to use the device or if the device did not work. Table 2 shows the key benefits and concerns mentioned by the PAB members regarding the remote monitoring solutions previously described in addition to any other particular feature they would like to be included.

**Table 2:** Open Questions and Feedback about potential benefits and concerns raised from people with dementia and caregivers.

**Benefits**	**Concerns**
**People with dementia**	
• I'd be interested in knowing what information is being collected • Great to know certain information/details about my health • Monitors heart rate and blood pressure • Beneficial to understand your sleep patterns, sleep quality or sleep duration • Makes your life considerably easier • Gives information about time, date, and alarm • The person should be able to receive SMS/texts • It should be simple and cute • Waterproof • Speak the messages and text to speak • GPS tracker and alarm SOS • “Locate/ Find your device” function if the person does not remember where the device is • Locate each other (e.g., I know where my wife is, and she knows where I am) • It should enable people to feel safe when they are on their own • Help me to find my way home • Map whilst cycling or being outside	• It could be intimidating (e.g., the camera) • Worried about maintaining human contact (don't want this to be replaced by devices) • The person may forget to charge the devices, misplace it or lose it • The person may forget how to use the device • The person may feel anxious if the device does not work, stops working, or the person has difficulties to make it work—ideally, someone should be able to access the device remotely and fix it for the person. • The device should be quite robust/solid • Soft material, not make the person sweaty • Color which the person likes • Compatible with other Apps that the person likes/uses • Adapted to native language of the user • Would it work in very cold weather (−32° in the winter in some countries)? • Rechargeable Battery • Can you charge it whilst wearing it? • How long does it take to charge? • Battery life • Device's guarantee?
**Caregivers**	
• Helpful to know that the person is being monitored • Peace of mind—Reduces worries • Gives people independence • Enables people to live alone • It may help to save money (e.g., linked to delayed institutionalization) • Believe to be beneficial after some time	• Having to be a bit knowledgeable to it • Too expensive • Would not want to be observed (myself)

## Discussion

This PI activity allowed a great deal of interaction between the PAB members who are “experts by experience” and the researchers. The different features any device might have were also presented to them and ordered by personal importance. In this way, the researchers were able to identify and extract preferences and features absolutely important to carry on the selection. The PAB members were presented with different varieties of wrist-worn devices. With assistance from caregivers and researchers when needed, they were asked to rate the various features and to order them by personal preference. The device names were not disclosed for the sake of simplicity and to avoid bias. Thus, the researchers were not bound to select the specific devices presented in the meeting, but rather have extracted guidelines and preferences to select from the enormous pool of ever-changing devices in the market and literature. Exploring users' rating of wearable solutions helps to understand users' requirements and preferences for e-health services and provide suggestions for e-health system construction. The present PI activity aimed to explore the main factors affecting wearable sensors and particular features and application characteristics acceptable to people with dementia and their caregivers while exploring their preferences. The feedback provides insightful design implications for e-health developers, clinicians and service providers, and several key findings can be derived from this work. From their feedback, activity level, HR, and respiratory rate as well as practical elements such as appearance, battery life, and the device being waterproof were all relevant aspects to consider. The latter, seemed particularly relevant in the case of dementia due to cognitive impairments and possible stigmatization (in the case of appearance).

To the best of our knowledge, this is one of the first PI activities having explored the perceptions, priorities, and concerns of people with dementia and their caregivers from different countries regarding remote monitoring solutions and wearable sensors. In accordance with previous studies (Wang et al., 2011; Deng et al., 2014; Liu and Yang, 2014; Peek et al., 2014; Calvillo et al., 2015; Khosravi and Ghapanchi, 2015; Yusif et al., 2016; Hoque and Sorwar, 2017; Alshahrani et al., 2019), PAB members underlined the potential of using the technology for daily monitoring particular aspects such as Heart and Respiratory Rate as well as Sleep quality and daily activity while they stated that they would like to receive certain information—report about the health status and metrics which is consistent with (Steggell et al., 2011; Liu et al., 2016; Klemets et al., 2019). However, none of the proposed features presented was clearly rejected but rated lower compared to the others (e.g., distance count, calories). Feedback from the PAB showed that signaling emergencies (emergency button) was rated highly (as the most preferable feature by the 19% of the participants) and indicated in open questions that they would find it beneficial. Concerns were raised surrounding the topic of battery life, since it was prioritized as the second most important metric. This result is in alignment with previous studies which support that from technical perspective, short battery life was one of the main issue for technology adoption by older people (Chen et al., 2012; Sun and Rau, 2015; Jamal et al., 2016). The wearables in this study range from 1- to 7-day battery life, depending on their heavy or light use of sensors and their purely research-oriented to more lifestyle-oriented purpose. Currently wearables are reaching close to 14-day battery life and prototypes of no-charge self-powered wearables, e.g., using thermoelectric energy to convert body heat to electricity, are emerging. According to the findings, such future developments would highly facilitate assisted living research and practice.

Several PAB members expressed also some critical concerns with regard to privacy issues of handling data from caregivers and clinicians, which is in line with (Claes et al., 2013, 2015; Peek et al., 2014). Similarly to other studies, financial costs have been identified as a major concern of people with dementia and their caregivers regarding wearable sensors and remote monitoring technologies (Chen et al., 2012; Xue et al., 2012; Claes et al., 2015; Sun and Rau, 2015). This is in line with the present paper's findings, in which the caregivers explicitly indicated that they would be reluctant to pay high costs for such devices by themselves. Moreover, it was highlighted also that the water-resistance is of high importance since the people with dementia may not be able to remember to remove it before taking a bath or washing their hands. Also, feedback suggested that some people with dementia may be more willing to accept technology that supports them in their daily functioning, in addition to assessing it. For example many referred to GPS and in particular, to a feature which would help them to track the route back home as very important, This indicated that they would value a digital device which would be useful for the researchers but also to them (e.g., to manage finding their way home and dealing with orientation problems, which is a common problem in people with dementia). Also, the caregivers believed that one of the core benefits from using such technology would be the delay in institutionalization since they would feel safer to monitor their relatives with dementia. Also, our PI activity revealed that personalization, appearance, degree of usefulness, and ease of use would be factors contributing to acceptance of technology, a finding compatible with previous research (Wu and Wang, 2005; Wu et al., 2007; Tung et al., 2008).

According to the recent systematic reviews exploring the factors influencing the adoption of the technology by older people, among the top common barriers in the adoption of technology by older people is the familiarity and access, need for assistance, trust, privacy implications, design, reduced dexterity, precision, and physical issues (e.g., hearing loss), the cost of the device, forgetting how to operate technology, false alarms and how to turn them off, obtrusiveness, low ease of use, potential negative effect on health, loss of control over technology and stigmatization, functionality and suitability for daily use, perception of no need, fear of dependence, limited training tailored to older learners, feeling of embarrassment, autonomy, loss of dignity, and social inclusion (Fischer et al., 2014; Peek et al., 2014; Claes et al., 2015; Khosravi and Ghapanchi, 2015; Sun and Rau, 2015; Liu et al., 2016; Yusif et al., 2016; Zhao et al., 2018; Alshahrani et al., 2019). Similar to the aforementioned studies, the present PI activity revealed that the appearance and style of the remote monitoring technology is of high importance to them. Moreover, based on existing studies, the benefits of using technology include safety, perceived usefulness, independence, and reduced “burden” on family caregivers, perceived need, monitoring their health status, social influence, influence of family and friends and professional caregivers (Peek et al., 2014). However, the strong, positive acceptance in the present PI activity indicates that PAB members might be willing to adopt wearable monitoring technology. Moreover, the participants considered one device (Bracelet 1) as being the optimal solution, highlighting as main features its screen showing daily feedback, its battery lasting for days and its affordable cost (less than EUR 150). These findings are vital since understanding factors of technology acceptance plays a pivotal key role to device selection for trials and research and, later on, the successful adoption of solutions and services based on technology (Wilkowska and Ziefle, 2009).

## Conclusion

The present PI activity indicated that PAB participants were in general willing to accept and incorporate remote monitoring technologies based on wearable devices into their daily lives. Furthermore, various concerns and requirements related to the use, battery life, features to be extracted, functioning and financing of the monitoring devices have to be considered, since they might hinder acceptance of the technology. To the best of our knowledge, no prior PI activities have investigated different perspectives among several people in dementia and pre-dementia stages as well as caregivers across different countries in Europe. Moreover, it has been conducted through face-to-face contact and not by telephone interviews, where time length is a critical limitation. In addition, the PAB had the opportunity to explore the particular features of each device hands-on, to interact with them and have their features and metrics explained to maximize their potential to understanding them and their potential benefits and pitfalls. A systematic way for the PAB to provide feedback in a straightforward and measurable manner was devised using cards, to rate and order features and devices by preference. By considering their feedback, future research design and clinical practice, researchers, technology developers as well as policy makers, and professional caregivers can promote the acceptance and implementation of remote monitoring in the care of people with dementia. As the PI activity was conducted in the framework of the RADAR-AD research project, its valuable insights were already used to support important decisions related to its ongoing developments and mainly the choice of devices to be used in prospective European remote monitoring cohort study with research participants from pre-dementia to dementia stages. A positive impact on the recruitment, retention and wellbeing of the RADAR-AD research participants is expected, demonstrating the importance of PI in dementia research.

## Data Availability Statement

The data that support the findings of this PI activity are available on request from the corresponding author, TS. The data are not publicly available since they contain information that could compromise the privacy of PI activity participants.

## Ethics Statement

Written informed consent was obtained from the relevant individuals for the publication of any potentially identifiable images or data included in this article.

## Author Contributions

TS, IL, AD, DG, and JG: conceptualization, validation, and investigation. TS and IL: methodology, formal analysis, writing – original draft preparation, and visualization. TS, SN, and IK: software. CH, MT, and IK: resources. TS, IL, NM, and EP: data curation. AD, DG, JG, NM, EP, and SN: writing – review and editing. JG, NM, EP, CH, MT, SN, and IK: supervision. TS, JG, NM, EP, CH, MT, SN, and IK: project administration. TS, JG, NM, EP, CH, MT, and IK: funding acquisition. All authors have read and agreed to the published version of the manuscript.

## Conflict of Interest

EP was employed by company Alfasigma Schweiz AG. NM is an employee of Janssen Research and Development, LLC and holds equity. The remaining authors declare that the research was conducted in the absence of any commercial or financial relationships that could be construed as a potential conflict of interest.

## Footnotes

^1^About the PAB: https://www.radar-ad.org/patient-engagement/patient-advisory-board/about-pab.

^2^The RADAR-AD Project: https://www.radar-ad.org/.

^3^https://www.radar-ad.org/patient-engagement/patient-advisory-board/general-members-pab.

^4^https://www.invo.org.uk/wp-content/uploads/2016/05/HRA-INVOLVE-updated-statement-2016.pdf.
